# Enrichment of leukocytes in peripheral blood using 3D printed tubes

**DOI:** 10.1371/journal.pone.0254615

**Published:** 2021-07-23

**Authors:** Li-fang Guo, Liu Wang, Sai Ren, Ning Su, Kun Wei, Xian-Ge Sun, Xiao-Dong Ren, Qing Huang

**Affiliations:** 1 Department of Laboratory Medicine, Army Medical Center, Chongqing, P.R. China; 2 Department of Laboratory Medicine, Southwest Hospital, Army Medical University, Chongqing, P. R. China; 3 Faculty of Materials and Energy, Institute for Clean Energy & Advanced Materials, Southwest University, Chongqing, China; Massachusetts Institute of Technology, UNITED STATES

## Abstract

Leukocytes have an essential role in patient clinical trajectories and progression. Traditional methods of leukocyte enrichment have many significant limitations for current applications. It is demonstrated a novel 3D printing leukocyte sorting accumulator that combines with centrifugation to ensure label-free initial leukocyte enrichment based on cell density and size. The internal structure of leukocyte sorting accumulator (revealed here in a new design, leukocyte sorting accumulator-3, upgraded from earlier models), optimizes localization of the buffy coat fraction and the length of the period allocated for a second centrifugation step to deliver a higher recovery of buffy coats than earlier models. Established methodological parameters were evaluated for reliability by calculating leukocyte recovery rates and erythrocyte depletion rates by both pushing and pulling methods of cell displacement. Results indicate that leukocyte sorting accumulator-3 achieves a mean leukocytes recovery fraction of 96.2 ± 2.38% by the pushing method of layer displacement. By the pulling method, the leukocyte sorting accumulator-3 yield a mean leukocytes recovery fraction of 94.4 ± 0.8%. New procedures for preliminary enrichment of leukocytes from peripheral blood that avoid cellular damage, as well as avert metabolic and phase cycle intervention, are required as the first step in many modern clinical and basic research assays.

## Introduction

Enumeration of leukocyte cells within peripheral blood provides valuable clinical information to physicians about the status of a patient. Leukemia and HIV can both be diagnosed and monitored by assessing leukocyte sub-populations [1]. These examples, as well as many others, have led to the development of new creative methods to produce and use the information about leukocytes to predict patient clinical trajectories and progression, as well as to guide treatment modalities on patient-to-patient basis [2]. As possibly the most clinically-relevant cell population in whole blood, the proportion of leukocyte cells in blood cells is very small [3]. Therefore, the novel enrichment leukocytes methods that reduce the costs and analysis time for isolating and characterizing leukocytes are important to delivering modern clinical medicine to large population groups.

The traditional approaches for label-free enrichment of leukocytes from whole blood are density gradient centrifugation (DGC) and cell lysis buffer [4,5]. DGC relies on the different cell densities to produce separation, which is typically accomplished with a density gradient medium like Ficoll, Percoll, sucrose, or dextran [6,7]. For example, Ficoll density centrifugation is used to separate leukocytes and circulating tumor cells (CTC) from the remainder of whole blood [8]. However, Ficoll density centrifugation requires milliliters of sample, is time- and labor-intensive, and requires the use of trained personnel and different density gradient mediums. Lysis buffers utilizes different reagents that lyse cells by imposing unbalanced osmotic gradients like NaCl and NH_4_Cl buffer to selectively lyse erythrocytes, which is the most common protocol for sample preparation for flow cytometry (FCM) [9]. Though it has many virtues, many studies report that false-positive results are obtained when surface markers for monocytes were examined using the lysis method. These errors were attributed to the phenomenon called Fcγ receptor (FcγR)-mediated trogocytosis [10]. Physical or chemical stress during isolation represents additional stimuli that can also activate leukocytes [11]. The Fluorescence-activated cell sorting (FACS) and magnetic-activated cell sorting (MACS) have been intensely investigated as cell separation technologies which show high selectivity for the target cells, however, such antibody labeling is not easily reversed [12]. In addition, only limited types of cells can successfully be separated by antibodies, because antibodies may trigger different signals that initiate a range of permissible cell pathways, such as apoptosis [3]. Centrifugal blood cell separation technology is the most widely used in clinical practice [13]. Its basic principle is based on the different size and density of blood cells. Although this method can achieve the purpose of enrichment leukocytes, the purity and concentration of leukocytes are low.

The emergence of three-dimensional (3D) printing technology provides a new way to deal with these problems. Recently, the microdevices processed by 3D printing technology have grown exponentially, demonstrating the great potential for cell biology, medical laboratory science, gene diagnosis [14,15]. Compared with traditional processing technology, 3D printing technology have incomparable flexibility and accuracy [16]. Several 3D printing materials have biocompatibility, which can meet the research and application needs of biological sciences and medicine [17]. Meanwhile, the manufacturing cost of 3D-printing technology is cheap, which has a very positive significance for the application and promotion of disposable biomedical microdevices [18,19]. Although 3D-printing technology has been widely used in the field of medicine, it is rarely reported about enrichment of human peripheral blood cells.

To enrich morphology and activity human leukocyte cells in peripheral blood, the study demonstrates a novel biomedical microdevice that is based on 3D-printing technology, which is identified as a leukocyte sorting accumulator (LSA). Under certain centrifugation conditions, according to different sedimentation rates of blood cells, the special internal structure of LSA have achieved the separation of buffy coat and erythrocyte layer, and finally bring about the purpose of enriching leukocytes. The novel biomedical microdevice does not use any chemical reagents (media density gradient or antibodies) which can act as a label-free initial leukocyte enrichment. In this study, established methodological parameters of the leukocyte sorting accumulator 3.0 (LSA-3) have improved enrichment efficiency, which results in enhancement in the leukocytes recovery rate above 96.20 ± 2.38% and 94.4 ± 0.8%, respectively. The corresponding mean erythrocyte depletion rate is 92.90 ± 2.52% and 91.2 ± 1.3% using either the pushing or pulling style method for isolating the buffy coat layer. Fifty-one samples of clinical blood have been measured.

## Materials and methods

### Blood samples preparation

Whole blood samples from 51 healthy volunteers were collected in Vacutainer blood tubes containing EDTA anti-coagulant (BD Bio- Sciences) at the Army Medical Center. All subjects in this study provided written informed consent. The study was approved by the Medical Ethics committee of Army Medical Center in accordance with relevant guidelines and regulations. Generally, 2–3 ml of whole blood was withdrawn from each healthy volunteer within an hour of the start of each experiment.

### Design of the leukocyte sorting accumulators

**Fig 1:**
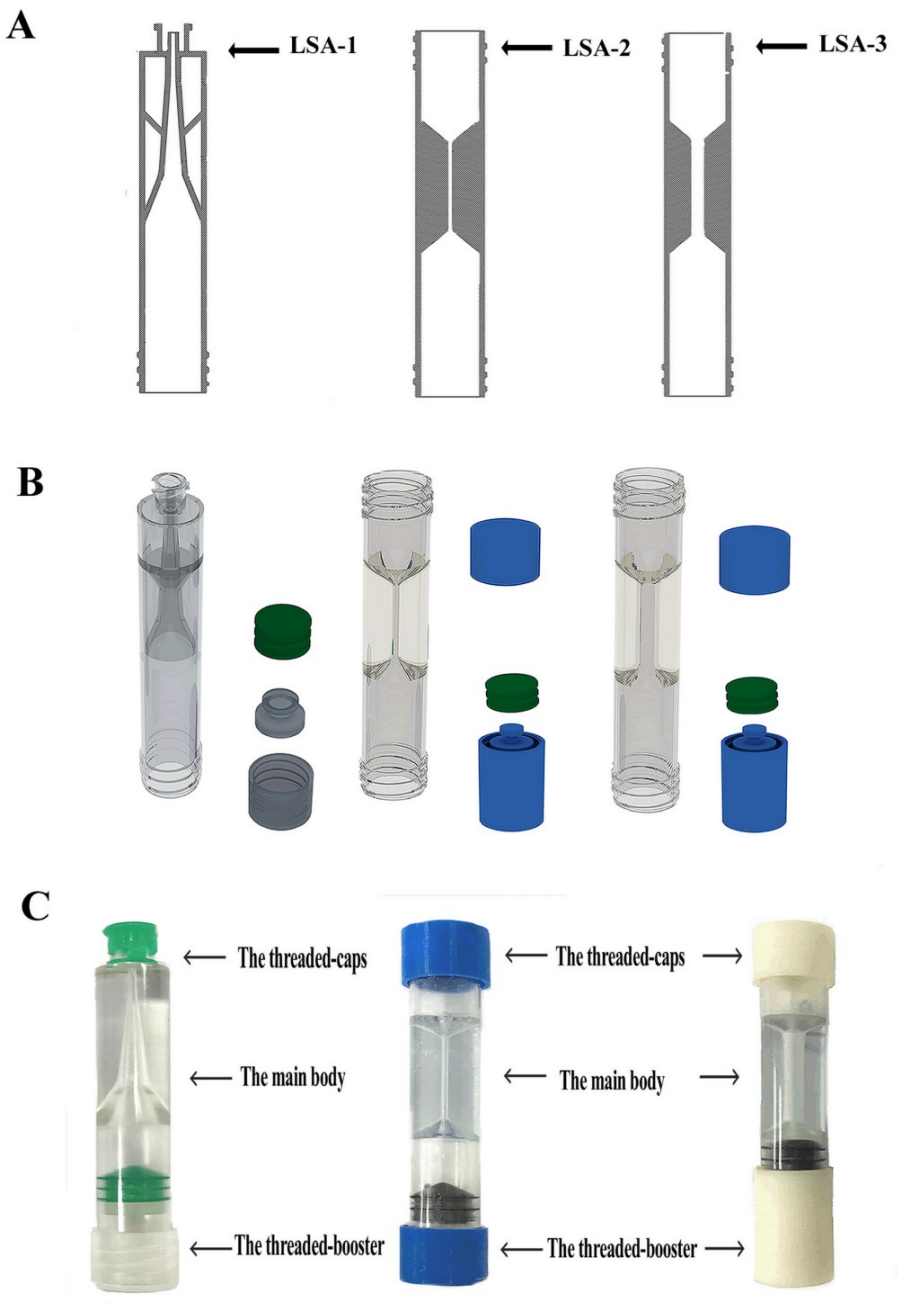
Schematic diagram of the leukocyte sorting accumulators. (A) Drawings of the LSAs models (from left to right: LSA-1, LSA-2 and LSA-3). (B) 3D modeling of the LSAs. (C) Photographs of the LSAs. LSA models consisting of the threaded-caps, the main body, and the threaded-booster.

The structure of leukocyte sorting accumulator (LSA) have an overall uniform length of 70 mm and a diameter of 12 mm, including the main body, the threaded-booster, and the threaded-cap (Fig 1). For the leukocyte sorting accumulator 1.0 (LSA-1), the graphic structure design of the main body takes the shape of hollow-like cone, similar in structure to a trumpet (Fig 1C, left). The outlet of the LSA-1 which located at the narrow end of the trumpet can be connected to syringe conical fittings with a 6% Luer taper (GB/T1962.1, China). The threaded-booster and the bottom of the main body are intersecting by the thread of high precision 3D-printing to ensure good airtight and prevent leaking. The black parts right above the threaded-booster is a “rubber ring” which is from 5ml syringe (GB15810-2019, China). The sealing property of threaded-booster with “rubber ring” is superior to that of without “rubber ring”. It includes the upper funnel, the central channel, and the lower funnel in the main body of leukocyte sorting accumulator 2.0 (LSA-2) and leukocyte sorting accumulator 3.0 (LSA-3, Fig 1B). The key difference between the LSA-2 and LSA-3 is the diameter of the central channel. The function of the central channel is to connect to the upper and the lower funnel chamber to achieve blood cell exchange, and its diameter directly affects the efficiency of blood cell exchange. Besides, the purpose of threaded-booster is to move clockwise and anticlockwise freely on the main body similar to screws and nuts. It is very ingenious to clockwise rotate the threaded-booster of the main body to cause the blood moving relatively from the lower to the upper funnel of the LSAs models under positive pressure. The same principle also exists in anticlockwise rotating of the threaded-booster to bring about the liquid counter moving under negative pressure.

### 3D printed the leukocyte sorting accumulator

In this study, LSA models were 3D printed according to the procedure described below. The LSA designs were laid out using AutoCAD software (Auto desk, San Rafael, Fig 1A). 3D modeling and part visualization were accomplished using 3D Studio Max software (Autodesk 3Ds Max, 2012, Fig 1B). We contracted Ming-jian Wei-ye Technology Co., Ltd. (Chongqing, China) to fabricate LSAs models consisting of the threaded-cap, the main body and the threaded-booster through utilizing UV laser cure of photopolymers by well-established high precision Stereo Lithography Appearance (SLA) system technology (Lite300HD, Union Tech, USA). The main body of LSAs models were fabricated by propylene polymer as 3D-printing material, with good biocompatibility, nontoxicity and excellent clarity, permitting observation of the liquid movement. The threaded-cap and threaded-booster were fabricated by Polyimide (PI) which is cheaper than other 3D-printing materials.

### Enrichment of leukocytes by the leukocyte sorting accumulator

We tested the three types of LSA design with samples consisting of 2–3 ml of the undiluted whole blood that were introduced into the main chamber to enrich leukocyte. The first centrifugation experimental conditions are set up as 2500 g for 10 min at room temperature (in a Beckman Allegra^®^ X-5 centrifuge, USA), and the second centrifugation step is set up as 2500 g for 2 min at room temperature. The LSAs designs display good tightness from the assembly of the threaded-booster, the main body and the threaded-cap. The whole process does not require antibodies or density gradient media, which is a label-free cell sorting and size-based separations technology.

### 1. Enrichment of leukocytes by the LSA-1 designs

1. Firstly, the LSA-1 design was assembled, and the sample was loaded in the chamber (S1A and S1B Fig).
2. The buffy coat formed when the sample was processed in the first centrifugation step (S1C Fig).
3. A syringe conical is next connected to the outlet of the LSA-1. Plus, with a anticlockwise rotation to push the threaded-booster of the main body, you can see clearly that most of the plasma and buffy coat is transferred to the syringe under positive pressure (S1D Fig).
4. Finally, it is collected and cells were counted on automatic hemocytometer, including erythrocyte cell and leukocyte cell count.

### 2. Enrichment of leukocytes by the LSA-2 prototype

1. The LSA-2 is assembled, the blood is introduced in the chamber (S2A and S2B Fig).
2. A significant difference exist among individuals with normal-range of hematocrit, so that the buffy coat was formed at an uncertain position of the lower funnel after the first centrifugation step (S2C Fig).
3. The buffy coat is going to move relatively from the lower to the upper funnel of the LSA-2 under positive pressure by anticlockwise rotating the threaded-booster of the main body (S2D Fig).
4. It is collected cells from the upper funnel for complete blood cell count.

### 3. The pushing style method of leukocyte enrichment by the LSA-3 design

**Fig 2:**
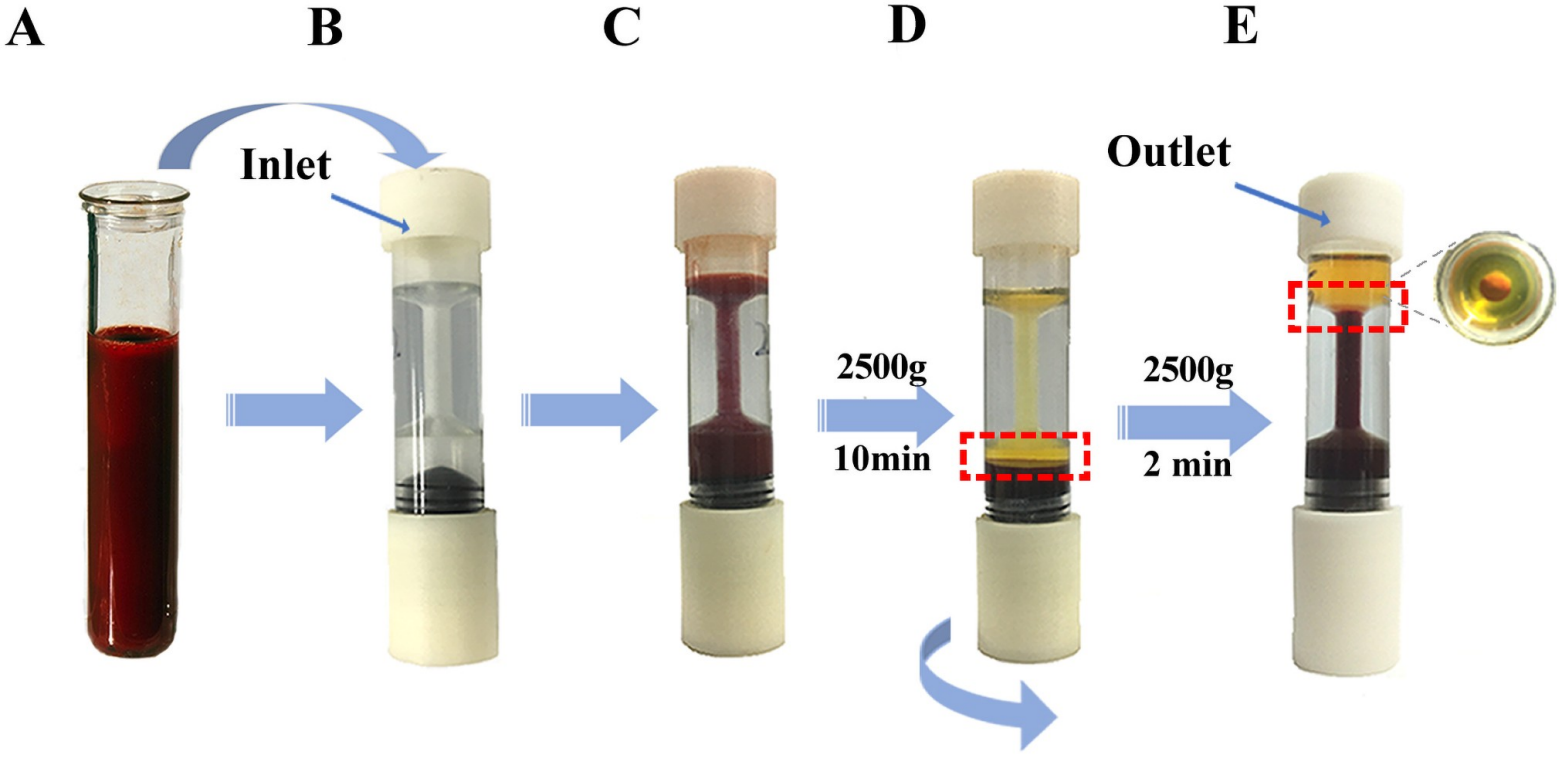
Processing of leukocyte extraction by LSA-3 the pushing style method. (A) 2–3 ml of whole blood was being prepared. (B, C) The sample is filling in the lower funnel chamber. (D) The buffy coat (red rectangle) was formed by centrifuging at 2500g for 10min. (E) with anticlockwise rotating the threaded-booster of the main body, the buffy coat layer (red rectangle) responds by moving up to the upper from the lower funnel.

1. The sample is filling in the lower funnel chamber (Fig 2A–2C).
2. It is clearly that the buffy coat was formed at an uncertain position of the lower funnel for the first centrifugation step (Fig 2D).
3. with anticlockwise rotating the threaded-booster of the main body, the buffy coat layer responds by moving up to the upper from the lower funnel under the positive force (Fig 2E).
4. If the observed shape of the buffy coat layer is intact on the upper funnel, it will be collected into an empty tube for complete blood cell count. Otherwise, it is necessary to ensure integrity of the buffy coat by the second centrifugation condition.

### 4. The pulling style method of leukocyte enrichment by the LSA-3 design

**Fig 3:**
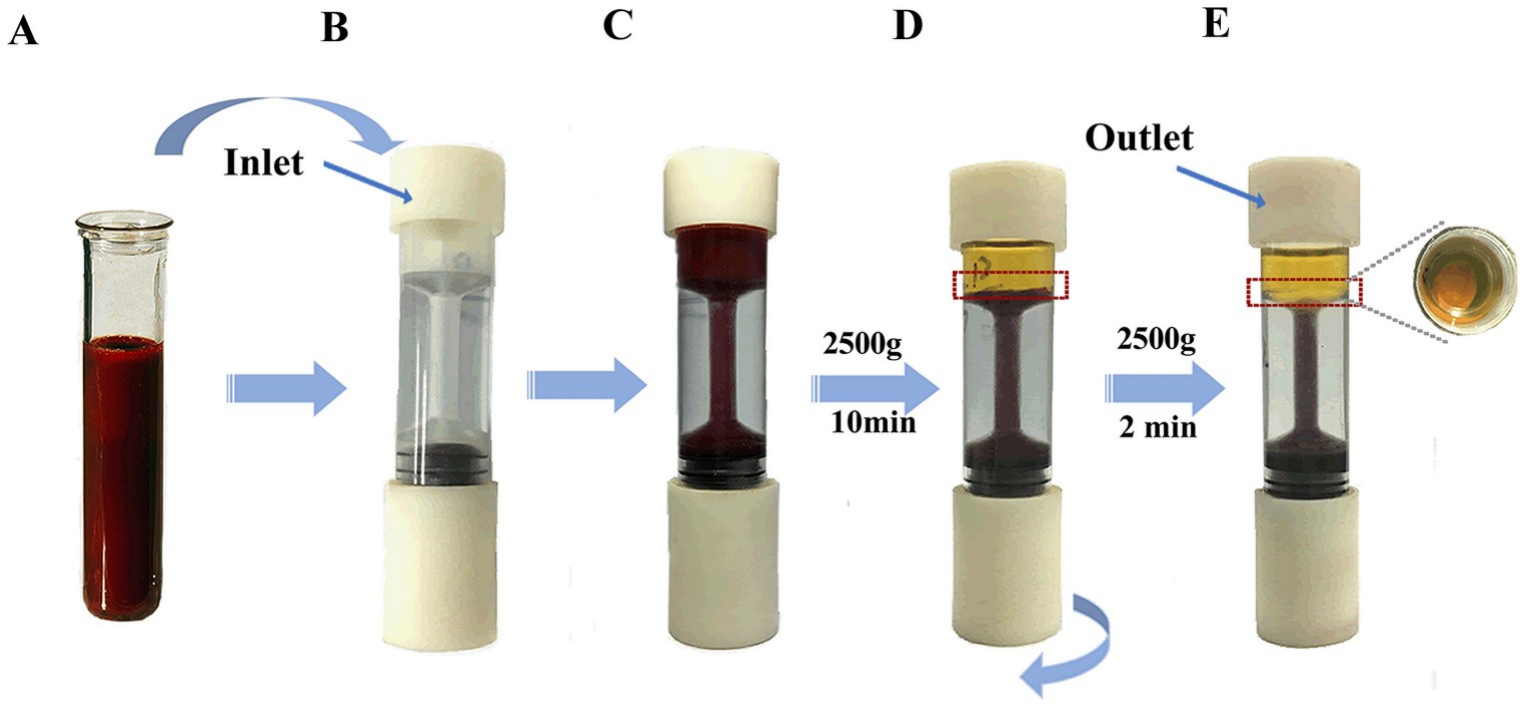
Processing of leukocytes by LSA-3 designs pulling style method. (A) 2–3 ml of whole blood was being prepared. (B) The LSA-3 is assembled. (C) the blood is introduced in the upper funnel chamber. (D) The buffy coat (red rectangle) is formed at 2500g for 10min centrifugation conditions. (E) with clockwise rotate the threaded-booster cause the buffy coat (red rectangle) moving down to a specific location within the upper funnel.

1. The LSA-3 is assembled, and the blood is introduced in the upper funnel chamber (Fig 3C).
2. The buffy coat is formed at an uncertain position of upper funnel at the first centrifugation conditions (Fig 3D).
3. It is very ingenious to clockwise rotate the threaded-booster of the main body to cause the buffy coat moving down to a specific location within the upper funnel under negative pressure (Fig 3E).
4. If the buffy coat layer has been broken up in the process, the second centrifugations will be required to coalesce the layer. Finally, it is collected from the upper funnel that cells were counted on automatic hemocytometer, including erythrocyte cell and leukocyte cell count.

### Optimization of pushing and pulling protocols by the LSA-3 device for enrichment of leukocytes

**Fig 4:**
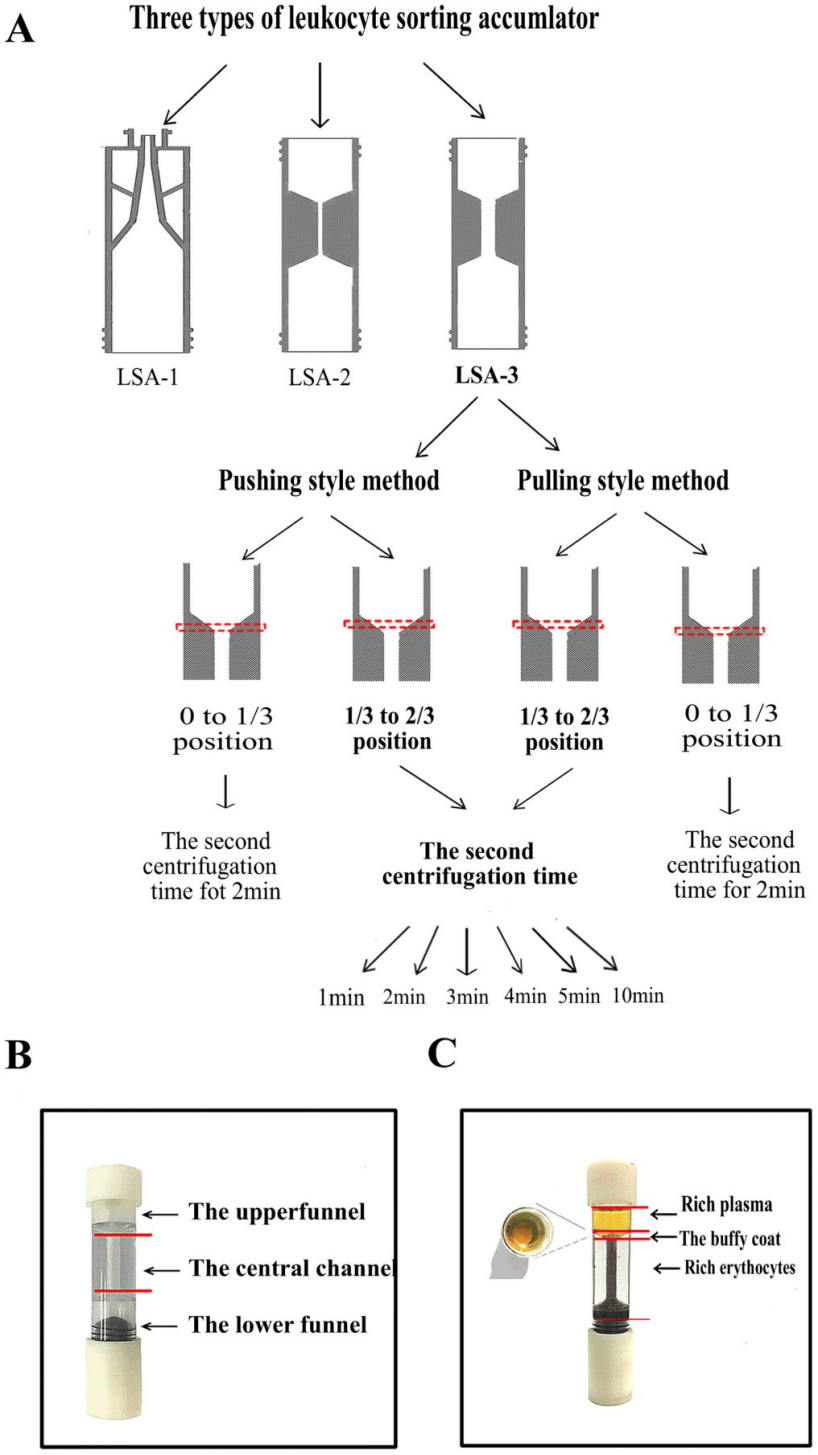
Schematic illustration of the working principles of the LSAs. (A) Flow-process diagram for enrichment of leukocytes by the LSAs models. The position of buffy coat in the upper funnel is identified by the red rectangle. (B) 3D printed LSA-3 including the upper funnel, the central channel and the lower funnel. (C) Whole blood separated by the cell density in three fractions: Rich platelets, the buffy coat and rich erythrocytes.

The study further considered the effect of position of the buffy coat layer in the upper funnel when the LSA-3 device is used and a second centrifugation step is required, followed by either a pushing style or pulling style of translocation. The buffy coat layer locates at the identified position in the upper funnel (either lower one-third segment (0 to 1/3) or the middle one-third (1/3 to 2/3) on LSA-3 (Fig 4A). When the buffy coat layer locates at the upper funnel between 1/3 to 2/3, whether the pushing or pulling method is employed, the second centrifugal time is set to range from 1 ~ 5, 10 min. Other experimental conditions and data analysis are the same as those described for LSA-1 above.

### Traditional centrifugal blood cell

As a control experiment, the blood cells were centrifuged at 2500g for 10min. Under certain centrifugation conditions, the sedimentation rate of blood cells is also different, from top to bottom are plasma layer, buffy coat and erythrocyte layer. The buffy coat was collected into a tube by pipette and counted on automatic hemocytometer.

### Cell lysis

Isolation of total leukocytes using erythrocyte lysis buffer was performed by mixing 1 ml of whole blood with 14 ml of lysis buffer (NH_4_Cl buffer) for 5 min. The mixture was then spun at 200 g for 4 min; the supernatant was discarded, and the pellet was resuspended in 1x PBS prior to staining.

### Wright–Giemsa staining

Morphological evaluation was accomplished using Wright-Giemsa staining (Sangon, China), in which most of erythrocytes had previously been depleted by novel LSA-3 pushing style methods. As a control, erythrocytes of sample had previously been lysed using cell lysis buffer and the suspension was stained using Wright-Giemsa. The prepared slides were inspected under a microscope. Cells that showed clear cytoplasm were considered viable using Wright-Giemsa staining (magnification, x40).

### Flow cytometry

Cells was then analyzed by flow cytometry to measure viability. Cells were deal with standard cell lysis in a tube labeled tube 2. Cells were treated with LSA-3 pushing style methods in a tube labeled tube 3. Then, cells were washed in five volumes of RPMI-1640 medium with FBS to maintain cell viability and centrifuged at 250 g for 10 min and resuspended in 2 mL RPMI -1640 medium in. A volume of 0.5 mL of the each cell suspension was diluted with 1.5 mL RPMI -1640 medium. A volume of 0.5 mL of this original sample was incubated for 30 min with -20°C ice-cold RPMI-1640 medium in a separate tube labeled tube 1 which act as a negative control. The tubes 1, 2 and 3 were incubated at 37° C for 10 min, centrifuged at 250 g for 10 min and resuspended in 100 μL AnnV binding buffer at a final concentration of 1×10^4^ cells/mL (BD Pharmigen, Mississauga, Canada). 7-actinomycin D (7AAD) (BD Pharmigen) were added in combination into tubes 1, 2 and 3, and incubated at room temperature for 20 min and analyzed with a FC500 flow cytometer (Beckman Coulter).

### Cell count and data analysis

The novel biomedical microdevice has been developed by our laboratory to collect the leukocyte, the approach uses the above protocol. The whole plasma fraction occupying the layer above the buffy coat is collected to the syringe of LSA-1 and to the upper funnel of LSA-2 and LSA-3. The volume of the buffy coat layer of LSA-1 can measure its volume directly in the syringe, and of LSA-2 and LSA-3 devices can measure by transferring its to an empty tube. A complete blood count is performed in triplicate with an automated hemocytometer, including the plasma fraction above the buffy coat count, such as platelets, lymphocytes, monocytes, granulocytes and erythrocytes. We also collected all remaining cells in the LSAs devices like the above procedures.

This is experimental data from a complete blood count as performed on an automated instrument. Leukocyte recovery rate (%) is calculated using the following equation:

$$\text{Leukocyte}\;\text{recovery}\;\text{rate}\left(\%\right)=N_{up}/N_{t}=N_{up}/(N_{up}+N_{down}).$$ (1)

Here, *N*_*up*_ refers to the populations of leukocyte in the upper funnel of the LSAs. *N*_*down*_ represents the number of leukocyte cell after removal of the upper funnel volume from LSAs device. *N*_*t*_ represents the sum of *N*_*up*_ and *N*_*down*_. The erythrocyte depletion rate (%) was calculated by a similar equation:

$$\text{Erythrocyte}\;\text{depletion}\;\text{rate}\left(\%\right)=M_{up}/M_{t}=M_{up}/(M_{up}+M_{down}).$$ (2)

Where the *M*_*up*_ represents the number of erythrocytes in the upper funnel of the LSAs and *M*_*down*_ refers to the number of erythrocyte cells after removal of the upper funnel volume from LSAs device. Data were collected in an Excel spreadsheet and then transferred and analyzed with the IBM SPSS Statistics Package for Windows, Version 21.0 (Armonk, NY: IBM Corp). Data from replicated experiments are reported as the mean values ± SD. Differences in mean values derived between LSA-3 by the pushing method and pulling method were compared using the t-test evaluation of the probability of the null hypothesis and by a one-way ANOVA among three or more groups. A value of *p < 0*.*05* was considered significant in all cases.

## Results

### The principle of LSA-3 device

The principle of LSA-3 is presented below, together with a preliminary review of the procedure for separation of the buffy coat from blood components. Under certain centrifugation conditions, the sedimentation rates of blood cells are different, from top to bottom are rich platelets, the buffy coat and rich erythrocytes [20,21] (Fig 4B and 4C). The leukocyte cells distribute in the buffy coat along a density profile. Because of the significant difference among individuals within normal-range hematocrit, the buffy coat was located in the middle one-third (1/3 to 2/3) position of the upper funnel after clockwise or anticlockwise rotate of the threaded-booster of LSA-3. The upper funnel is designed as an inverted vertebral body. As the diameter of the bottom of the upper funnel (either lower one-third segment (0 to 1/3) or the middle one-third (1/3 to 2/3) becomes smaller and smaller, the buffy coat is concentrated at the bottom and visible to the naked eye, which can greatly reduce the residual volume of erythrocytes at the bottom of the upper funnel. As the result, erythrocyte cells are removed as much as possible without affecting the retention of the buffy coat to improve the purity of leukocytes. Further, it is the main function that the central channel can connect the upper and the lower funnel chamber to realize blood cell exchange, and its diameter directly affects the resistance of blood cell exchange. Therefore, the diameter of LSA-3 is larger than LSA-2 in the central channel so that the resistance of the cell exchange under centrifugal force becomes smaller than that of the LSA-2 design.

In this procedure, this method does not require antibodies or density gradient media in the whole process, which can act as a label-free cell sorting and size-based separations, thus producing preliminary enriched buffy coats containing some platelets and rich leukocyte suspensions depleted of most erythrocytes. The LSA-3 design is an upgrade from LSA-1 and 2, which optimizes the geometric structure of in the main body to deliver a higher recovery of buffy coats than earlier designs.

### Enrichment of leukocytes by the LSAs

**Fig 5:**
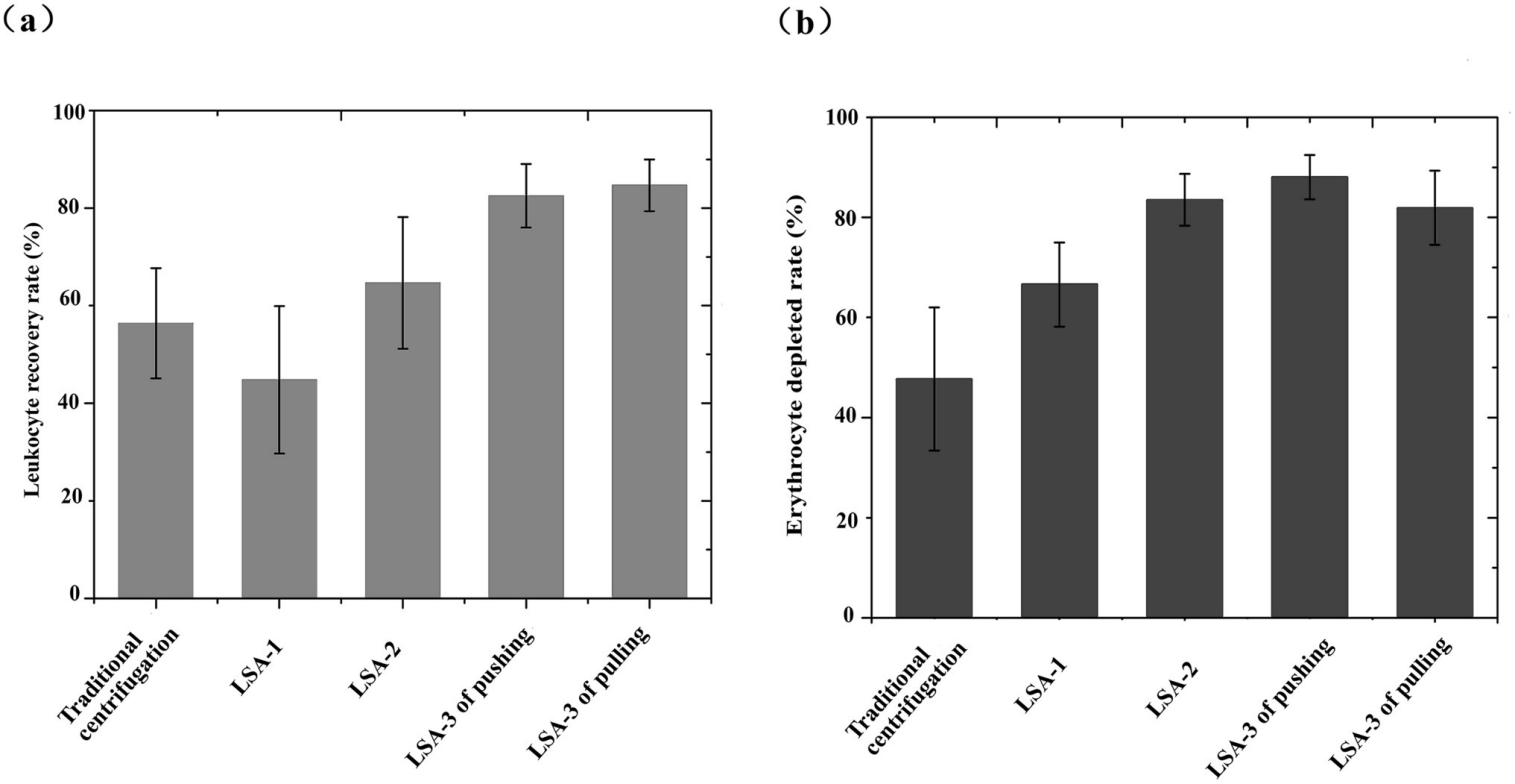
Enrichment of leukocytes and depletion of erythrocytes by the LSAs. (A) The mean leukocytes recovery rate of the LSAs and traditional centrifugal blood cell. (B) The mean erythrocyte depletion rate of the LSAs and traditional centrifugal blood cell.

Compared with traditional centrifugal blood cell, the LSA-3 yields a significantly improved population of buffy coat cells at its outlet. The mean leukocyte recovery yield from traditional centrifugal blood cell, the LSA-1, the LSA-2 and LSA-3 by pushing and pulling methods for advancing cell layers were LSA-1: 44.8 ± 15.1% (*p = 0*.*034*); LSA-2: 64.7 ± 13.5% (*p = 0*.*029*); LSA-3: pushing 82.5 ± 6.5% (*p = 0*.*028*); LSA-3 pulling 84.7 ± 5.3% (*p = 0*.*019*), traditional centrifugal blood cell: 56.4 ± 11.3% (*p = 0*.*023*), respectively (Fig 5A). These mean values were significantly higher when cell sorting was achieved by the LSA-3 pushing and pulling methods of cell displacement than were obtained from other methods, with a clear discriminative difference seen in the mean values. The mean erythrocyte depletion rate in traditional centrifugal blood cell method, LSA-1, LSA-2, and LSA-3 that utilize pushing and pulling methods of cell enrichment were LSA-1: 66.6 ± 8.4% *(p = 0*.*35*); LSA-2: 83.5 ± 5.2% (*p = 0*.*289*); and LSA-3 pushing: 88.03 ± 4.4% (*p = 0*.*46)*, LSA-3 pulling: 81.9 ± 7.4% (*p = 0*.*54*) and traditional centrifugal blood cell:47.4 ± 14.3% (*p = 0*.*026*), respectively (Fig 5B). These mean values of the traditional method were significantly different to the other groups. The result shows that the mean erythrocyte depletion rate in the traditional centrifugal blood cell is lower than the former four groups. We found significant improvements through use of the most effective geometry of LSA-3 compared with the LSA-1, the LSA-2 and traditional centrifugal blood cell. Therefore, follow-up studies only focused on using the LSA-3 designs to produce a high recovery of leukocytes.

### Optimizing the methodological parameters of leukocyte enrichment by LSA-3

The result of the preliminary LSA-3 experiments show that the position of the buffy coat layer locates at heights between the lower one-third segment (0 to 1/3) or the middle one-third (1/3 to 2/3) segment in the upper funnel, which would make a difference of the efficiency of leukocytes recovery. Upon discovering that the buffy coat lands in the lower third (0 to 1/3) segment of the upper funnel, using of the pushing style method the leukocyte recovery rate was found to be 85 ± 4.9% (see Table 1, *p = 0*.*009*). Under these circumstances, use of the pulling method of extraction was found to produce comparable recovery to the pushing method, with little significant difference between the two groups (Table 1, *p = 0*.*2501*).

**Table 1:** Optimizing the methodological parameters of leukocyte enrichment by LSA-3.

	Pushing style		Pulling style	
	Leukocyte recovery rate (%)	Erythrocyte depleted rate (%)	Leukocyte recovery rate (%)	Erythrocyte depleted rate (%)
0 to 1/3 position				
Mean	85	95.4	86.5	89.5
SD*	4.9	3.5	2.89	4.69
1/3 to 2/3 position				
Mean	96.20	92.90	94.4	91.2
SD*	2.38	2.52	0.8	1.3

*SD standard deviation. The 0 to 1/3 position refers to the lower one-third segment in the upper funnel on the LSA-3. The 1/3 to 2/3 position represents the middle one-third segment in the upper funnel on the LSA-3.

The mean leukocyte recovery rate using both the pushing and pulling methods of displacement was found to be significantly higher when the buffy coat layer landed in the middle 1/3 to 2/3 segment of the upper funnel (96.2 ± 2.38% recovery by pushing, 94.4 ± 0.8% recovery by pulling). Both the pushing and pulling methods produced mean capture values that were significantly higher for buffy coats landing in the middle 1/3 to 2/3 segment than in the lower third 0 to 1/3 segment. No differences were found in the mean erythrocyte depletion rates when the buffy coat layer localized at either the lower third (0 to 1/3) or middle third (1/3 to 2/3) position in the upper funnel (Table 1, *p = 0*.*3011*), and no significance difference with respect to using the pulling vs pushing methods were found between the two groups (Table 1, *p = 0*.*2918*).

When the buffy coat locates at the middle third (1/3 to 2/3) position in the upper funnel, cell enrichment methods of pushing and pulling were found to produce no statistical difference in the mean leukocyte recovery fractions between the two groups (Table 1, *p = 0*.*0992*). Therefore, we conclude that buffy coat localization at the middle third (1/3 to 2/3) position of the upper funnel is the most significant and useful signature for efficient enrichment of leukocytes. This signature is therefore preferred over buffy coat localization in the middle third (1/3 to 2/3) position of the upper funnel for cell enrichment by either the pulling or pushing methods.

### Effect of imposing a second centrifugation step on refining recovery yields

We measured the recovery and depletion yields when a second centrifugation step was used to coalesce the buffy coat layer after the layer was originally displaced by pushing and pulling action. Differences in recovery and depletion fractions were measured. When the buffy coat locates at the middle third (1/3 to 2/3) position of the upper funnel after using the pushing method to adjust cell displacement, the mean leukocyte recovery rate was found to be 94.4 ± 1.1%, 96.2 ± 2.4%, 95 ± 0.02%, 95.5 ± 0.01%, 93.5 ± 0.02%, and 96.7 ± 1%, respectively, after executing a second centrifugal step lasting either 1 min, 2 min, 3 min, 4 min, 5 min, or 10 min. These mean values were not statistically different after 2 min of centrifugation compared to the other five groups of second centrifugation intervals (*p = 0*.*175*, *p = 0*.*415*, *p = 0*.*267*, *p = 0*.*258*, *and p = 0*.*163* for 1 min, 3 min, 4 min, 5 min, and 10 min, respectively). In comparison, the use of the pulling method for buffy layer displacement to the middle third (1/3 to 2/3) position of the funnel produced mean recovery values of 89.2 ± 1.3%, 94.4 ± 0.5%, 91.1 ± 3.14%, 94.9 ± 1.2%, 92.3 ± 2.9%, and 95.0 ± 3.5%, respectively, after second centrifugation times of 1 min, 2 min, 3 min, 4 min, 5 min, and 10 min. These mean recovery values were not statistically different after 2 min of centrifugation compared to the other five groups (*p = 0*.*061*, *p = 0*.*276*, *p = 0*.*377*, *p = 0*.*167*, *p = 0*.*423* for 1 min, 3 min, 4 min, 5 min, and 10 min, respectively); this data is shown in S1 Fig.

In summary, we optimized the methodological parameters that determine maximal leukocyte recovery by localizing the buffy coat layer at the middle third segment (1/3 to 2/3) of the funnel regardless of whether the pushing or pulling method of extraction was employed. A second centrifugation step lasting 2min produces the best leukocyte recovery consistent with highest erythrocyte removal in the shortest time.

### Analysis of leukocyte morphology and activity after cell lysis and cell extraction by the LSA-3

**Fig 6:**
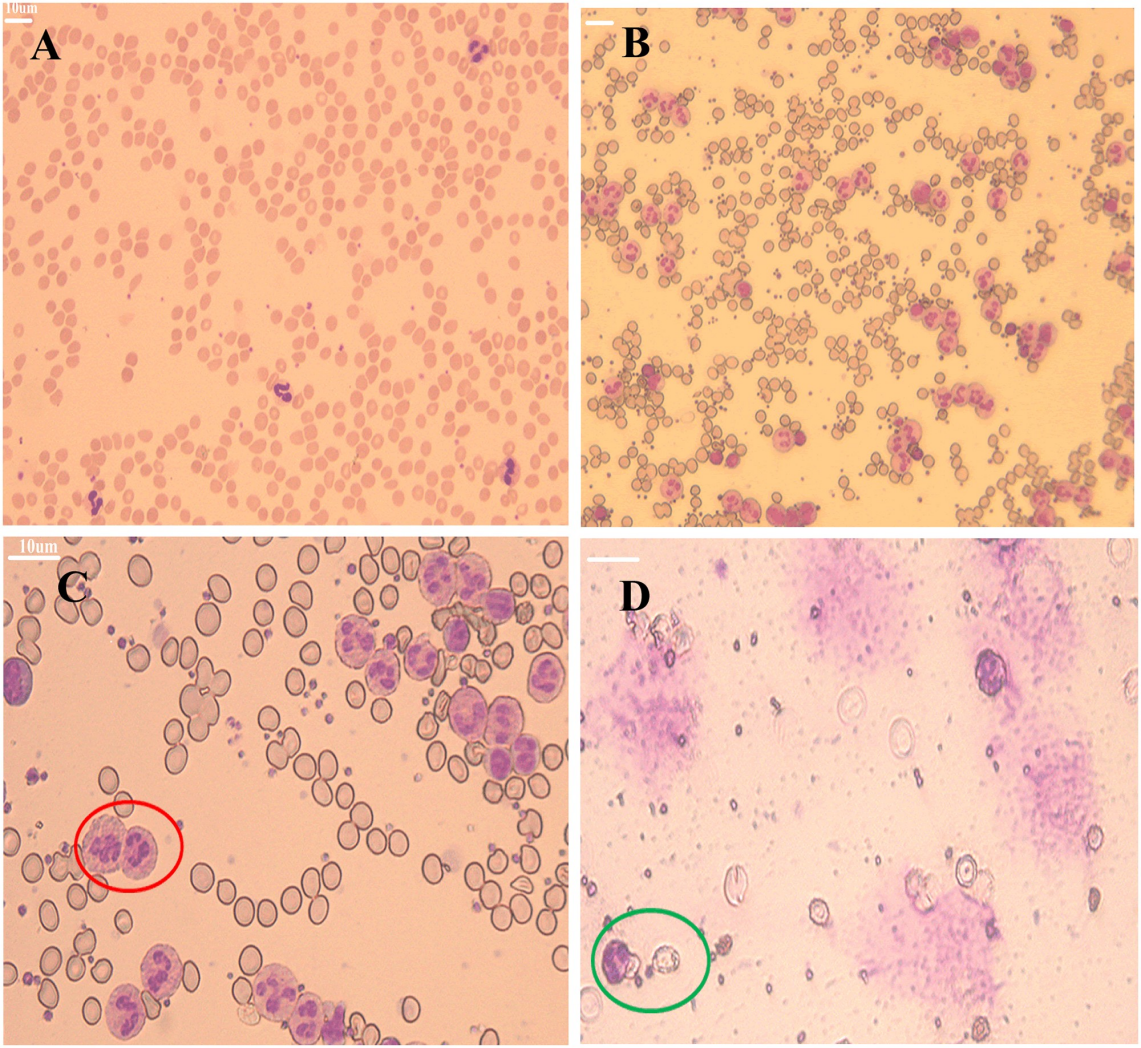
Morphology analysis of leukocytes respective after cell lysis and the LSA-3 pushing style method. (A) Wright-Giemsa staining of whole blood samples obtained using a 20x microscope objective. (B) The LSA-3 pushing style method of cell displacement. Leukocytes concentration has been significantly enhanced (20x objective). (C) Nucleated cells with clear cytoplasm after Wright-Giemsa staining are considered viable leukocytes (magnification, x40; representative leukocytes identified by red ellipse). (D) After cell lysis buffer treatment of whole blood cells, the leukocytes clear cytoplasm (indicating viability and homeostasis) was coated with rich erythrocyte debris (magnification, x40, identified by green ellipse).

Comparison of the blood samples obtained after pre- and post-processing by LSA-3 pushing style methods was assayed by Wright-Giemsa staining and shown in the representative micrographs of Fig 6A and 6B. It is observed under bright field illumination at 20-fold magnification. In this case, cell enrichment was achieved by LSA-3 pushing style methods. The mean leukocyte recovery rate was 96.2 ± 2.38%, and mean erythrocyte depleted rate was 92.90 ± 2.52%. Qualitatively, the results in Fig 6B show that leukocyte concentration has notably increased after treatment.

To determine if the processing of leukocytes for enrichment damage cells morphology, we analyzed the morphology of cells derived from the different isolation techniques. Cells were identified based on their morphology and differential staining with Wright-Giemsa staining. In Fig 6C, cells were enriched by the LSA-3 pushing style methods. It is very clear that leukocyte is labeled with a purple dye different from the erythrocyte cells. Cells with clear cytoplasm under 40x magnification after Wright-Giemsa staining were considered viable (red circle, Fig 6C). This has been considered a good indicator of viable cell morphology and homeostasis [22]. Leukocyte cells were treated with lysis buffer and stained with Wright-Giemsa. Cell lysis has been shown to produce approximately 100% recovery of leukocytes from blood samples and presence of erythrocyte debris (magnification, x40, green circle, Fig 6D). Meanwhile, the cytoplasm of leukocytes has become blurred, and cell morphology has already changed which maybe affect leukocytes initial physiological function. The staining results allow us to conclude that leukocytes have been completely maintained with a normal morphological structure by LSA-3 pushing style method.

In contrast to our findings with regard to the assessment of activity versus dead cells using 7AAD, the LSA-3 pushing style method (0.036 ± 0.038%, n = 3) treatment showed a very less effect on leukocyte cells compared to that on cell lysis (3.06 ± 0.76%, n = 3), confirming further that the cell lysis method predisposing leukocyte cells towards cell death under a hemolytic environment (S5 Fig). Also, we observed that negative control (tube 1) displayed a rapid increase in 7-AAD staining compared to study cases (tube 2 and tube 3).

### Clinical applications of leukocyte enrichment

Having established the enrichment of leukocytes by LSA-3, we explored the utility of adapting this approach to clinical practice. The results of 51 experiments with clinical blood samples consisting of 26 cases of cell enrichment using the pushing style method, as well as 25 cases using the pulling style method, were found to be consistent with the results of this study. The mean leukocyte recovery rate of the pushing and pulling methods is 95 ± 2.9% and 94 ± 2.5%, respectively. The corresponding mean erythrocyte depletion rate is 93 ± 2.7% and 91 ± 2.8% for the pushing and pulling methods, respectively (S2 Fig). The serial improvements in the design of the LSAs models result in an approximately 20-fold gain in the leukocyte yield of LSA-3, where the leukocyte-to-erythrocyte ratio increases from 1:1100 to 1:48 (S1 and S2 Tables). Specific conditions used to produce the enrichment data are described in S1 and S2 Tables in the Supporting information section.

## Discussion

The novel biomedical microdevice, utilizing centrifugation without introducing any chemical contamination (media density gradient or antibodies), significantly improves analysis of blood [23]. In comparison, methods used by other groups affect the separation of cells, and only cell lysis has been shown to produce approximately 100% recovery of leukocytes from blood samples [24]. However, the cytoplasm of leukocytes has become blurred, and cell morphology has already changed by cell lysis.

This novel and facile approach to blood sampling required optimization of several methodological parameters that were shown to affect cell capture efficiency. These include the LSA geometry design, the position at which the buffy coat localizes in the upper funnel, and the optimal duration of the second centrifugation. All these factors facilitate enrichment of leukocytes from whole blood. This work documents and validates the present device design and identifies the parameters that optimize separation and yield of homeostatic cells.

This technology provides new ideas and methods which was shown a label-free, size and density-based separation of blood cells with high throughput based on the localization of the buffy coat cells within a narrowly defined band occupying the upper funnel of LSA-3 designs. Based on these validation studies, the advantage of the present modifications to earlier LSA designs can be explained because of conserving extremely high leukocyte populations that were lost in earlier versions of the design. In LSA-1, Due to the outlet graphic structure of the main body taking the shape of a trumpet, leukocytes were found to adhere to the inner wall of narrow end the trumpet under the pressure applied during anticlockwise rotation the threaded-booster, resulting in failure to reach the enrichment target. To overcome this shortcoming in the design of LSA-1, we redesigned and fabricated LSA-2 by 3D-printing. Whole blood was naturally layered in LSA-2 by application of centrifugal force, and large numbers of leukocyte cells could pass through the central channel and to form the buffy coat within the outlet upper funnel. While this design showed improvement in the leukocytes recovery rate of LSA-2 over LSA-1, bottleneck jams of cellular traffic occurred because the diameter of the central channel of LSA-2. The central channel diameter of LSA-3 was larger than LSA-2 so that the resistance of the cell exchange was reduced, where an increased diameter of the central channel of the LSA-3 produced a significantly higher mean cell recovery rate and greater efficiency of cell separation.

Localization of the buffy coat layer within the outlet upper funnel after the first centrifugation step is important for achieving a maximal cell enrichment outcome. We found that a consistently greater enrichment can be achieved when the buffy coat localizes at the middle one-third (1/3 to 2/3) segment of the upper funnel of LSA-3 designs, as compared to localizing at the lower one-third segment of the outlet the upper funnel (the 0 to 1/3 position). With this criterion, high throughput and capture yield can be achieved by both the pushing and pulling methods. We speculate that in the earlier devices, a small number of leukocytes attached to the walls of the upper funnel, which caused cell loss.

In these studies, both the pushing and pulling methods of cell displacement in LSA-3 can be used to enrich leukocytes, with no significant difference in cell yields between the two options. The original intention of employing both methods was to let the researcher or clinician choose the method that was more workable and user-friendly. Based on the present studies, our results show that while a second centrifugal step is required to complete leukocyte isolation and transfer, its duration length does not have much effect on capture yields. Accordingly, we recommend that 2 min be allocated as the centrifugation time to accomplish optimal enrichment in the shortest time.

In summary, we report 3D printed several biomedical microdevices that perform label-free, high-throughput enrichment leukocytes from non-diluted whole blood by centrifugation. The novel LSA-3 microdevice utilizes 3D printing designs that improve enrichment efficiency by displacing the buffy coat layer farther away from the band of erythrocytes. The novel microdevice design requires no ancillary pumping mechanism nor expensive disposables to operate and may become a viable candidate for a standardized and streamlined initial isolation protocols in clinical laboratories. In recent years, the development of microfluidic technology is more and more rapid, but there are still many challenges, such as the interference of a large number of erythrocyte cells in the process of blood sample analysis [25]. Therefore, numerous assays require removal of erythrocytes from whole blood as an essential step to analysis of clinically-relevant cells. Such as CTC isolation by microfluidic devices [25], dielectrophoretic (DEP) devices [26], deterministic lateral displacement (DLD) [27]. The novel LSA-3 biomedical microdevice in this study accomplishes depletion of most erythrocytes from whole blood with good cell morphology and activity, which has the huge potential to be used as a valuable sample preparation tool in both research and clinical settings.

The novel LSA-3 biomedical microdevice also have some potential limitations. Future work will focus on improving the leukocyte-to-erythrocyte ratio and providing better standardize procedures for laboratory use. For example, further modifications of the diameter of the central channel may eliminate the initial dynamic reduction of leukocyte concentration caused by the impeded traffic of leukocytes during their entry into the upper funnel. In addition, height of the funnel could be altered to affect better separation efficiency of leukocytes. Furthermore, a higher height aspect of the funnel could result in a smaller residual volume of erythrocyte, thereby improving separation efficiency.

## Supporting information

S1 Fig Processing of leukocyte enrichment by LSA-1. (DOCX)

S2 Fig Processing of leukocyte enrichment by LSA-2. (DOCX)

S3 Fig The mean leukocytes recovery fractions achieved after varying the duration of the second centrifugation. (DOCX)

S4 Fig Leukocyte enrichment and erythrocyte depletion in 51 cases of LSA-3 fractionation using both the pushing and pulling methods of cell displacement. (DOCX)

S5 Fig Cells were harvested and processed for 7-AAD staining to assess cell death using flow cytometry. (DOCX)

S1 Table Clinical blood samples from 26 subjects tested in LSA-3 to produce enrichment of leukocytes by pushing style method. (DOCX)

S2 Table Clinical blood samples from 25 subjects tested in LSA-3 to produce enrichment of leukocytes using the pulling style method. (DOCX)

## References

[1] Alidjinou EK, Sane F, Lefevre C, Baras A, Moumna I, Engelmann I, et al. Enteroviruses in blood of patients with type 1 diabetes detected by integrated cell culture and reverse transcription quantitative real-time PCR. Acta diabetologica. 2017;54(11):1025–9. doi: 10.1007/s00592-017-1041-7 . 28861621

[2] Augustine TN, van der Spuy WJ, Kaberry LL, Shayi M. Thrombin-Mediated Platelet Activation of Lysed Whole Blood and Platelet-Rich Plasma: A Comparison Between Platelet Activation Markers and Ultrastructural Alterations. Microscopy and microanalysis: the official journal of Microscopy Society of America, Microbeam Analysis Society, Microscopical Society of Canada. 2016;22(3):630–9. doi: 10.1017/S1431927616000854 . 27329313

[3] Brinckmann M, Kaschina E, Altarche-Xifró W, Curato C, Timm M, Grzesiak A, et al. Estrogen receptor alpha supports cardiomyocytes indirectly through post-infarct cardiac c-kit+ cells. Journal of molecular and cellular cardiology. 2009;47(1):66–75. doi: 10.1016/j.yjmcc.2009.03.014 . 19341743

[4] Sun Y, Sethu P. Low-stress Microfluidic Density-gradient Centrifugation for Blood Cell Sorting. Biomedical microdevices. 2018;20(3):77. doi: 10.1007/s10544-018-0323-3 . 30155743

[5] Najar M, Rodrigues RM, Buyl K, Branson S, Vanhaecke T, Lagneaux L, et al. Proliferative and phenotypical characteristics of human adipose tissue-derived stem cells: comparison of Ficoll gradient centrifugation and red blood cell lysis buffer treatment purification methods. Cytotherapy. 2014;16(9):1220–8. doi: 10.1016/j.jcyt.2014.05.021 . 25065636

[6] Shiono H, Matsui T, Okada T, Ito Y. Single-step enrichment of basophils from human peripheral blood by a novel method using a Percoll density gradient. Journal of separation science. 2016;39(15):3062–71. doi: 10.1002/jssc.201600329 . 27293108 PMC4974159

[7] Lu Y, Ahmed S, Harari F, Vahter M. Impact of Ficoll density gradient centrifugation on major and trace element concentrations in erythrocytes and blood plasma. Journal of trace elements in medicine and biology: organ of the Society for Minerals and Trace Elements (GMS). 2015;29:249–54. doi: 10.1016/j.jtemb.2014.08.012 . 25240911

[8] Konczalla L, Ghadban T, Effenberger KE, Wöstemeier A, Riethdorf S, Uzunoglu FG, et al. Prospective Comparison of the Prognostic Relevance of Circulating Tumor Cells in Blood and Disseminated Tumor Cells in Bone Marrow of a Single Patient’s Cohort With Esophageal Cancer. Annals of surgery. 2019. doi: 10.1097/sla.0000000000003406 . 31188197

[9] Eidenschink Brodersen L, Menssen AJ, Wangen JR, Stephenson CF, de Baca ME, Zehentner BK, et al. Assessment of erythroid dysplasia by "difference from normal" in routine clinical flow cytometry workup. Cytometry Part B, Clinical cytometry. 2015;88(2):125–35. doi: 10.1002/cyto.b.21199 . 25490867

[10] Masuda S, Iwasaki S, Tomaru U, Sato J, Kawakami A, Ichijo K, et al. Mechanism of Fcγ receptor-mediated trogocytosis-based false-positive results in flow cytometry. PloS one. 2012;7(12):e52918. doi: 10.1371/journal.pone.0052918 . 23300821 PMC3531343

[11] Olwal CO, Ang’ienda PO, Onyango DM, Ochiel DO. Susceptibility patterns and the role of extracellular DNA in Staphylococcus epidermidis biofilm resistance to physico-chemical stress exposure. BMC microbiology. 2018;18(1):40. doi: 10.1186/s12866-018-1183-y . 29720089 PMC5930741

[12] Gray BP, Requena MD, Nichols MD, Sullenger BA. Aptamers as Reversible Sorting Ligands for Preparation of Cells in Their Native State. Cell chemical biology. 2020;27(2):232–44.e7. doi: 10.1016/j.chembiol.2019.12.004 . 31879266 PMC7038790

[13] Kambic HE, Nosé Y. Spin doctors: new innovations for centrifugal apheresis. Therapeutic apheresis: official journal of the International Society for Apheresis and the Japanese Society for Apheresis. 1997;1(3):284–305. doi: 10.1111/j.1744-9987.1997.tb00151.x . 10225752

[14] Lee VK, Dai G. Printing of Three-Dimensional Tissue Analogs for Regenerative Medicine. Annals of biomedical engineering. 2017;45(1):115–31. doi: 10.1007/s10439-016-1613-7 . 27066784 PMC5064823

[15] Zheng P, Hu X, Lou Y, Tang K. A Rabbit Model of Osteochondral Regeneration Using Three-Dimensional Printed Polycaprolactone-Hydroxyapatite Scaffolds Coated with Umbilical Cord Blood Mesenchymal Stem Cells and Chondrocytes. Medical science monitor: international medical journal of experimental and clinical research. 2019;25:7361–9. doi: 10.12659/MSM.915441 . 31570688 PMC6784681

[16] Kimijima T, Edanaga M, Yamakage M. Superior sealing effect of a three-dimensional printed modified supraglottic airway compared with the i-gel in a three-dimensional printed airway model. Journal of anesthesia. 2018;32(5):655–62. doi: 10.1007/s00540-018-2531-7 . 30022284

[17] Cruz RLJ, Ross MT, Powell SK, Woodruff MA. Advancements in Soft-Tissue Prosthetics Part B: The Chemistry of Imitating Life. Frontiers in bioengineering and biotechnology. 2020;8:147. doi: 10.3389/fbioe.2020.00147 . 32391336 PMC7191111

[18] Obregon F, Vaquette C, Ivanovski S, Hutmacher DW, Bertassoni LE. Three-Dimensional Bioprinting for Regenerative Dentistry and Craniofacial Tissue Engineering. Journal of dental research. 2015;94:143S–52S. doi: 10.1177/0022034515588885 . 26124216

[19] Legocki AT, Duffy-Peter A, Scott AR. Benefits and Limitations of Entry-Level 3-Dimensional Printing of Maxillofacial Skeletal Models. JAMA otolaryngology—head & neck surgery. 2017;143(4):389–94. doi: 10.1001/jamaoto.2016.3673 . 28056140

[20] Miron RJ, Chai J, Zhang P, Li Y, Wang Y, Mourão CFAB, et al. A novel method for harvesting concentrated platelet-rich fibrin (C-PRF) with a 10-fold increase in platelet and leukocyte yields. Clinical oral investigations. 2019. doi: 10.1007/s00784-019-03147-w . 31788748

[21] Ozer K, Kankaya Y, Çolak Ö. An important and overlooked parameter in platelet rich plasma preparation: The mean platelet volume. Journal of cosmetic dermatology. 2019;18(2):474–82. doi: 10.1111/jocd.12682 . 29862631

[22] Navas A, Giraldo-Parra L, Prieto MD, Cabrera J, Gómez MA. Phenotypic and functional stability of leukocytes from human peripheral blood samples: considerations for the design of immunological studies. BMC immunology. 2019;20(1):5. doi: 10.1186/s12865-019-0286-z . 30658588 PMC6339328

[23] Al Battah F, De Kock J, Ramboer E, Heymans A, Vanhaecke T, Rogiers V, et al. Evaluation of the multipotent character of human adipose tissue-derived stem cells isolated by Ficoll gradient centrifugation and red blood cell lysis treatment. Toxicology in vitro: an international journal published in association with BIBRA. 2011;25(6):1224–30. doi: 10.1016/j.tiv.2011.05.024 . 21645610

[24] Sun Y, Ren Y, Yang F, He Y, Liang S, Guan L, et al. High-yield isolation of menstrual blood-derived endometrial stem cells by direct red blood cell lysis treatment. Biology open. 2019;8(5). doi: 10.1242/bio.038885 . 31036750 PMC6550070

[25] Kamyabi N, Huang J, Lee JJ, Bernard V, Semaan A, Stephens B, et al. A microfluidic device for label-free isolation of tumor cell clusters from unprocessed blood samples. Biomicrofluidics. 2019;13(4):044111. doi: 10.1063/1.5111888 . 31462955 PMC6701978

[26] Hoettges KF, Henslee EA, Torcal Serrano RM, Jabr RI, Abdallat RG, Beale AD, et al. Ten-Second Electrophysiology: Evaluation of the 3DEP Platform for high-speed, high-accuracy cell analysis. Scientific reports. 2019;9(1):19153. doi: 10.1038/s41598-019-55579-9 . 31844107 PMC6915758

[27] Okano Hiromasa, Konishi Tomoki, Suzuki Toshihiro, Suzuki Takahiro, Ariyasu Shinya, Aoki Shin, et al. Enrichment of circulating tumor cells in tumor-bearing mouse blood by a deterministic lateral displacement microfluidic device. Biomedical Microdevices. 2015;17(3):59.1–11. doi: 10.1007/s10544-015-9964-7 26002773

