# Supplementary material for: Enrichment of leukocytes in peripheral blood using 3D printed tubes
Source: PLoS One. 2021 Jul 23;16(7):e0254615. doi: 10.1371/journal.pone.0254615 (PMC8301617; doi:10.1371/journal.pone.0254615)
Supplement: S1 Fig — (DOCX) [file pone.0254615.s001.docx]

**Fig S1. Processing of leukocyte enrichment by LSA-1.**


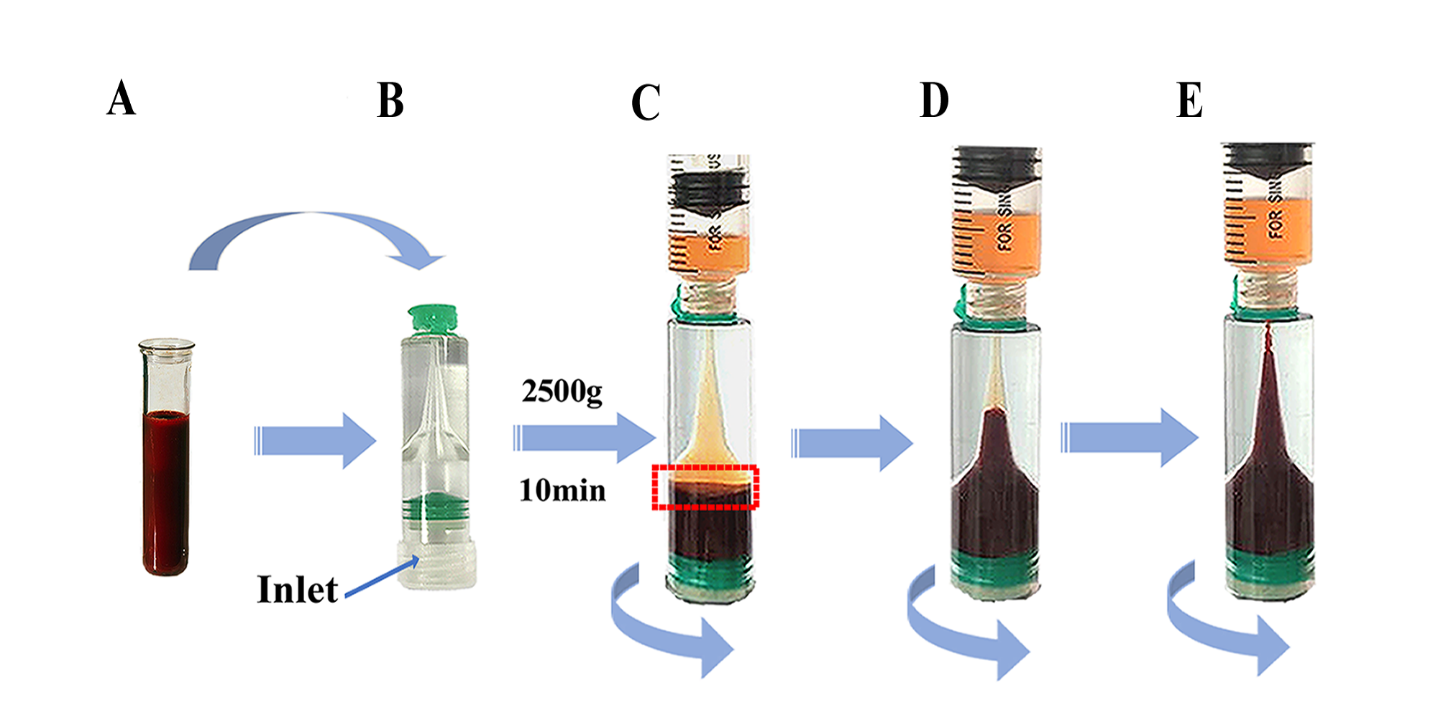


(A) 2–3 ml of whole blood was being prepared. (B) the LSA-1 designs were assembled, and the sample was loaded in the lower funnel chamber. (C) The buffy coat (red rectangle) was formed by centrifuging at 2500g for 10min. (D,E) The syringe conical is next connected to the outlet of the LSA-1. with a anticlockwise rotation to push the threaded-booster, most of the plasma and buffy coat is transferred to the syringe.
