# Supplementary material for: Enrichment of leukocytes in peripheral blood using 3D printed tubes
Source: PLoS One. 2021 Jul 23;16(7):e0254615. doi: 10.1371/journal.pone.0254615 (PMC8301617; doi:10.1371/journal.pone.0254615)
Supplement: S2 Fig — (DOCX) [file pone.0254615.s002.docx]

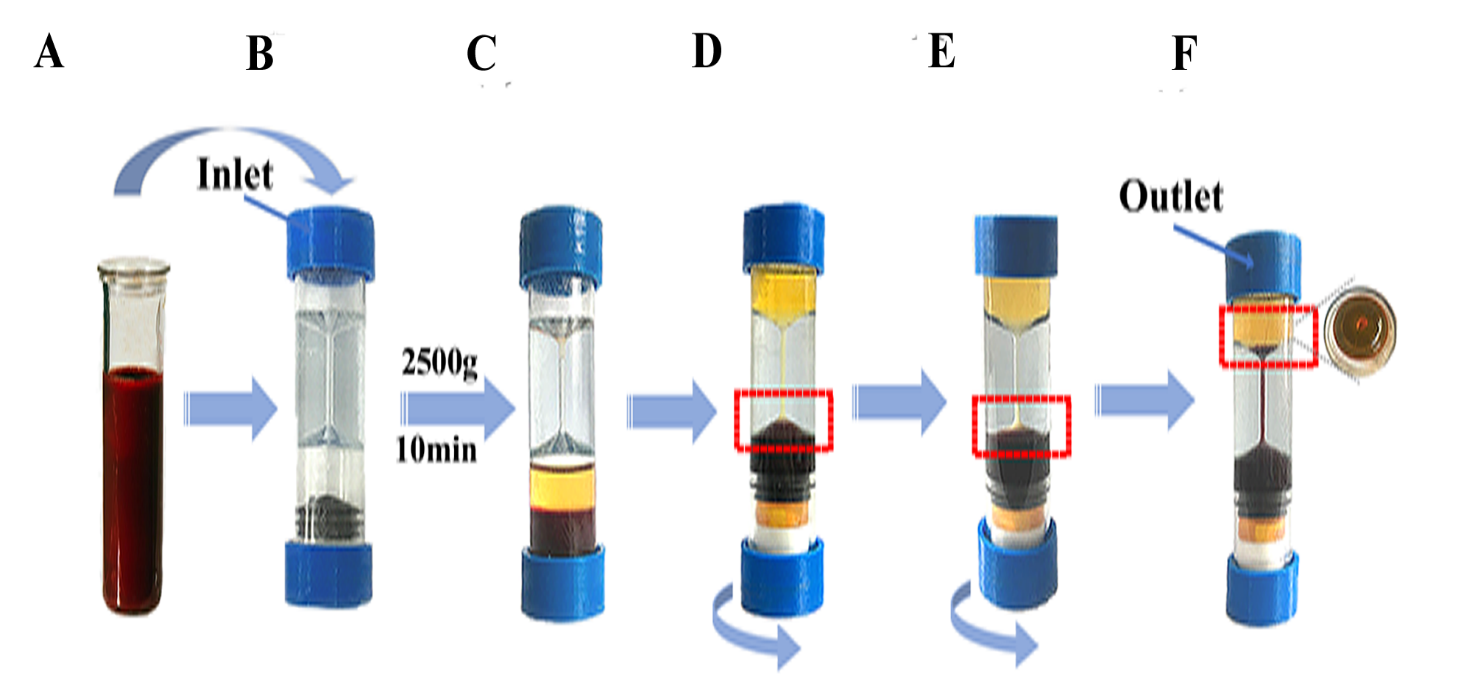
**Fig S2. Processing of leukocyte enrichment by LSA-2.**

(A) 2–3 ml of whole blood was being prepared. (B) the LSA-2 is assembled, the blood is introduced in the lower funnel chamber. (C) the buffy coat was formed at the lower funnel by the first centrifugation conditions. (D, E and F) The buffy coat(red rectangle) is going to move relatively from the lower to the upper funnel of the LSA-2.
