# Supplementary material for: Enrichment of leukocytes in peripheral blood using 3D printed tubes
Source: PLoS One. 2021 Jul 23;16(7):e0254615. doi: 10.1371/journal.pone.0254615 (PMC8301617; doi:10.1371/journal.pone.0254615)

**Fig. S3 The mean leukocytes recovery fractions achieved after varying the duration of the second centrifugation.**

#
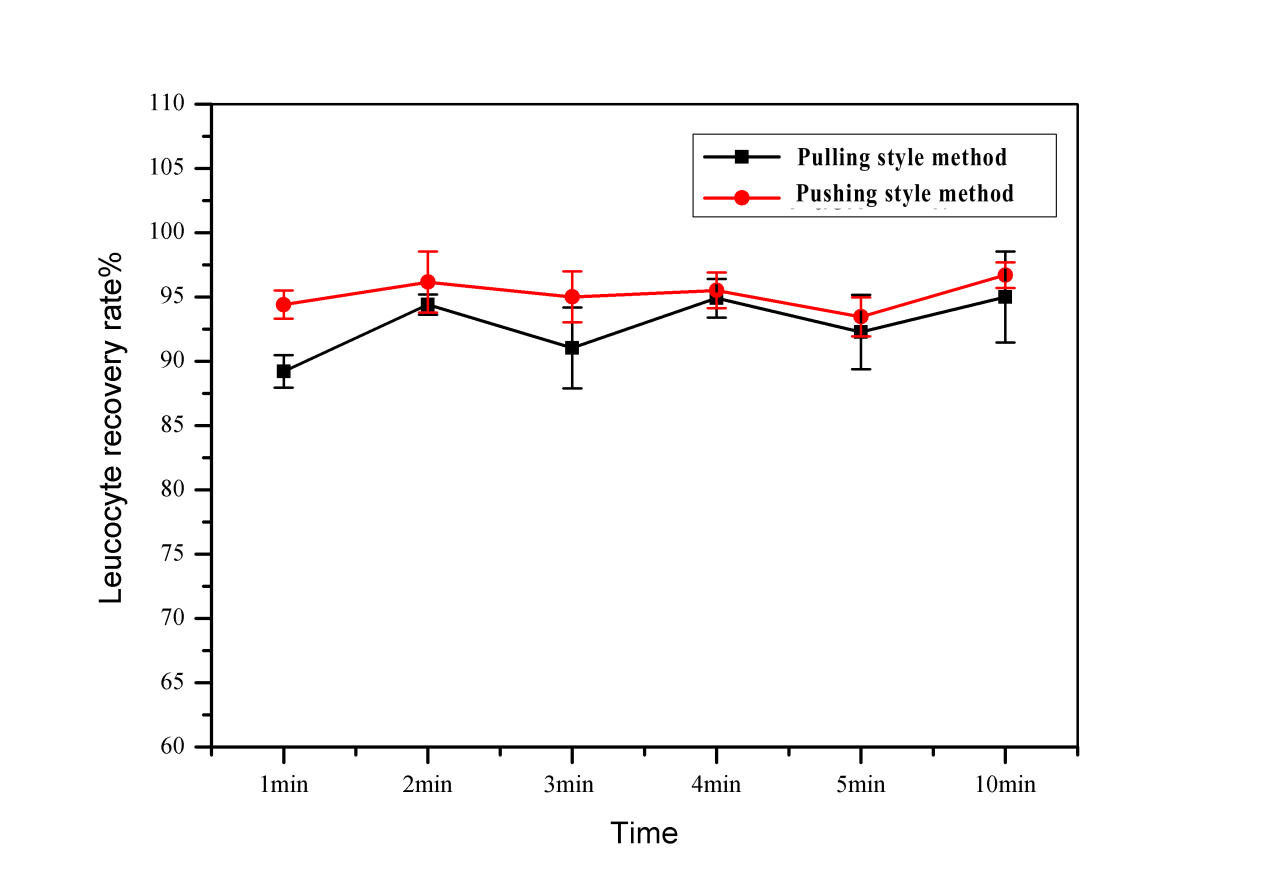

Supplement: S3 Fig — (DOCX) [file pone.0254615.s003.docx]
