# Supplementary material for: Enrichment of leukocytes in peripheral blood using 3D printed tubes
Source: PLoS One. 2021 Jul 23;16(7):e0254615. doi: 10.1371/journal.pone.0254615 (PMC8301617; doi:10.1371/journal.pone.0254615)
Supplement: S4 Fig — (DOCX) [file pone.0254615.s004.docx]

# Fig S4. Leukocyte enrichment and erythrocyte depletion in 51 cases of LSA-3 fractionation using both the pushing and pulling methods of cell displacement.

*

*

# The mean leukocyte recovery fractions achieved in LSA-3 by the pushing and pulling methods of cell displacement were 95 ± 0.03% and 94 ± 0.03%, respectively. Corresponding mean erythrocyte depletion fractions of 93 ± 0.02% and 91 ± 0.03%.
