# Supplementary material for: Enrichment of leukocytes in peripheral blood using 3D printed tubes
Source: PLoS One. 2021 Jul 23;16(7):e0254615. doi: 10.1371/journal.pone.0254615 (PMC8301617; doi:10.1371/journal.pone.0254615)
Supplement: S5 Fig — (DOCX) [file pone.0254615.s005.docx]

**
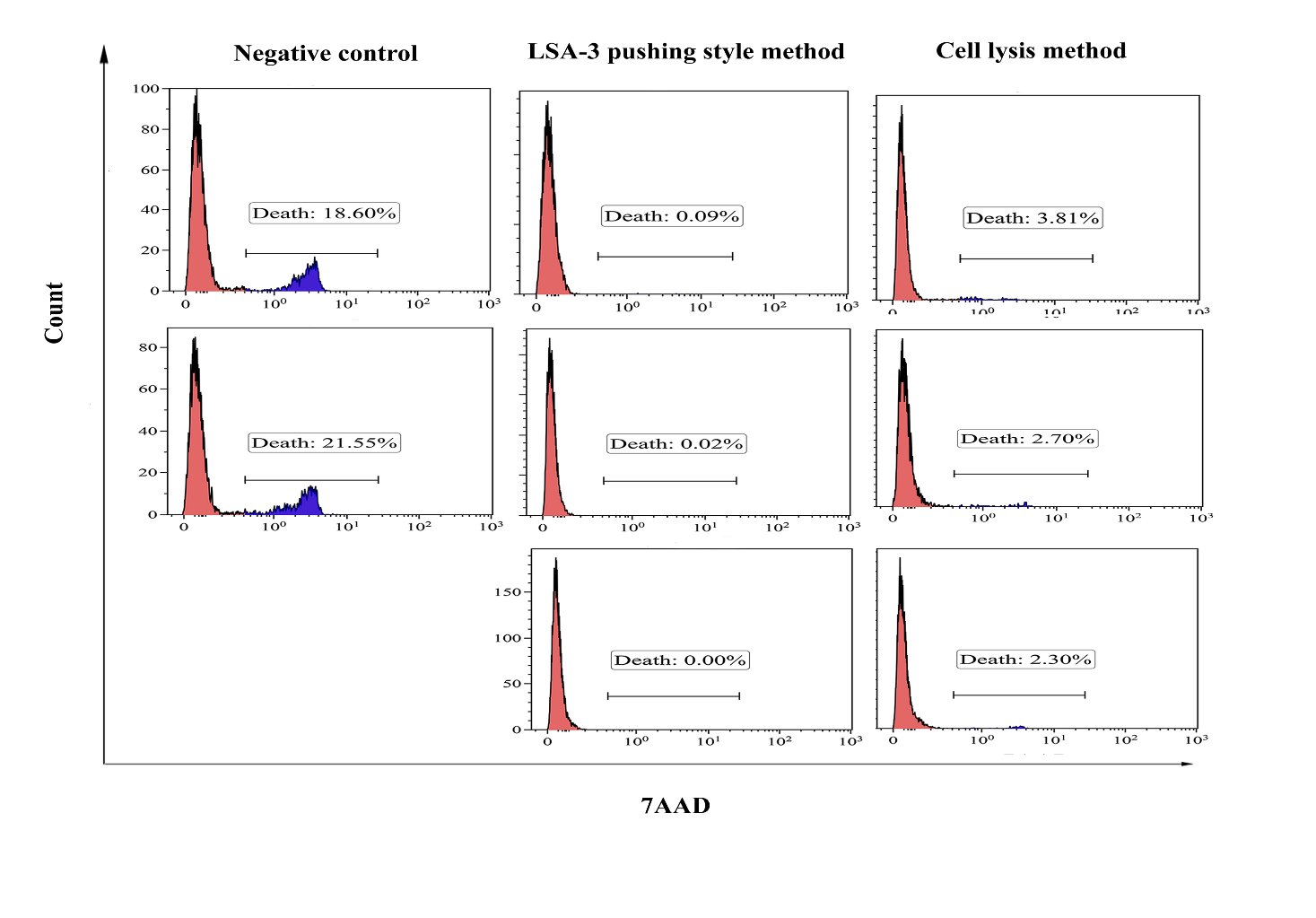
Fig S5. Cells were harvested and processed for 7-AAD staining to assess cell death using flow cytometry.**

Representative image from 3 independent experiments showing the percentage of 7-AAD-positive monocytes by negative control, LSA-3 pushing style method and cell lysis method, respectively.
