# Supplementary material for: Enrichment of leukocytes in peripheral blood using 3D printed tubes
Source: PLoS One. 2021 Jul 23;16(7):e0254615. doi: 10.1371/journal.pone.0254615 (PMC8301617; doi:10.1371/journal.pone.0254615)
Supplement: S1 Table — (DOCX) [file pone.0254615.s006.docx]

# Table S1. Clinical blood samples from 26 subjects tested in LSA-3 to produce enrichment of leukocytes by pushing style method.

| **ID** | **Sex** | **Total leukocyte cells (WBC *Nt* )** | **Total erythrocyt cells (RBC *Mt*)** | **Enrichment of leukocyte cells (WBC *Nup*)** | **Depletion of erythrocyte cells (RBC *Mdown*)** | **Leukocyte recovery rate (%)** | **Erythrocyte depleted rate (%)** | **Leukocyte-to-erythrocyte ratio** |
| --- | --- | --- | --- | --- | --- | --- | --- | --- |
| 1 | Male | 12.77×10^6^ | 6.54×10^9^ | 12.27×10^6^ | 6.17×10^9^ | 96% | 94% | 1:30 |
| 2 | Female | 7.18×10^6^ | 8.05×10^9^ | 6.71×10^6^ | 7.73×10^9^ | 93% | 96% | 1:47 |
| 3 | Female | 6.95×10^6^ | 6.31×10^9^ | 6.83×10^6^ | 6.04×10^9^ | 98% | 96% | 1:38 |
| 4 | Male | 11.08×10^6^ | 7.17×10^9^ | 10.41×10^6^ | 6.76×10^9^ | 94% | 94% | 1:39 |
| 5 | Male | 9.33×10^6^ | 5.99×10^9^ | 9.24×10^6^ | 5.33×10^9^ | 99% | 90% | 1:69 |
| 6 | Male | 8.45×10^6^ | 6.87×10^9^ | 8.05×10^6^ | 6.35×10^9^ | 95% | 92% | 1:60 |
| 7 | Female | 14.44×10^6^ | 7.71×10^9^ | 13.36×10^6^ | 7.40×10^9^ | 93% | 96% | 1:22 |
| 8 | Female | 15.45×10^6^ | 7.18×10^9^ | 15.02×10^6^ | 6.73×10^9^ | 97% | 94% | 1:30 |
| 9 | Male | 8.32×10^6^ | 8.60×10^9^ | 8.00×10^6^ | 8.20×10^9^ | 96% | 95% | 1:50 |
| 10 | Male | 14.95×10^6^ | 8.02×10^9^ | 14.28×10^6^ | 7.54×10^9^ | 96% | 94% | 1:33 |
| 11 | Female | 10.41×10^6^ | 6.63×10^9^ | 9.52×10^6^ | 6.28×10^9^ | 91% | 95% | 1:35 |
| 12 | Male | 8.97×10^6^ | 7.78×10^9^ | 8.60×10^6^ | 7.07×10^9^ | 96% | 91% | 1:81 |
| 13 | Female | 8.27×10^6^ | 6.72×10^9^ | 7.80×10^6^ | 6.08×10^9^ | 94% | 90% | 1:77 |
| 14 | Female | 9.06×10^6^ | 7.54×10^9^ | 8.83×10^6^ | 6.85×10^9^ | 97% | 91% | 1:70 |
| 15 | Male | 7.12×10^6^ | 8.44×10^9^ | 6.65×10^6^ | 8.18×10^9^ | 94% | 97% | 1:35 |
| 16 | Female | 7.41×10^6^ | 6.02×10^9^ | 6.95×10^6^ | 5.81×10^9^ | 94% | 97% | 1:30 |
| 17 | Male | 9.34×10^6^ | 6.88×10^9^ | 8.58×10^6^ | 6.60×10^9^ | 92% | 96% | 1:32 |
| 18 | Female | 9.50×10^6^ | 7.65×10^9^ | 9.13×10^6^ | 7.36×10^9^ | 96% | 96% | 1:30 |
| 19 | Male | 7.48×10^6^ | 6.80×10^9^ | 6.38×10^6^ | 6.47×10^9^ | 85% | 94% | 1:48 |
| 20 | Female | 7.84×10^6^ | 6.28×10^9^ | 7.78×10^6^ | 6.10×10^9^ | 99% | 97% | 1:22 |
| 21 | Female | 10.41×10^6^ | 5.73×10^9^ | 10.12×10^6^ | 5.06×10^9^ | 97% | 88% | 1:60 |
| 22 | Female | 10.69×10^6^ | 6.38×10^9^ | 10.43×10^6^ | 5.85×10^9^ | 98% | 92% | 1:48 |
| 23 | Female | 11.76×10^6^ | 5.74×10^9^ | 11.28×10^6^ | 5.17×10^9^ | 96% | 90% | 1:48 |
| 24 | Female | 12.68×10^6^ | 9.79×10^9^ | 12.47×10^6^ | 9.21×10^9^ | 98% | 94% | 1:44 |
| 25 | Female | 11.30×106 | 8.84×10^9^ | 10.82×10^6^ | 8.23×10^9^ | 96% | 93% | 1:55 |
| 26 | Female | 14.70×106 | 9.94×10^9^ | 13.70×10^6^ | 9.61×10^9^ | 93% | 97% | 1:23 |
