# Supplementary material for: Enrichment of leukocytes in peripheral blood using 3D printed tubes
Source: PLoS One. 2021 Jul 23;16(7):e0254615. doi: 10.1371/journal.pone.0254615 (PMC8301617; doi:10.1371/journal.pone.0254615)
Supplement: S2 Table — (DOCX) [file pone.0254615.s007.docx]

# Table S2. Clinical blood samples from 25 subjects tested in LSA-3 to produce enrichment of leukocytes using the pulling style method.

| **ID** | **Sex** | **Total leukocyte cells (WBC *Nt* )** | **Total erythrocyt cells (RBC *Mt*)** | **Enrichment of leukocyte cells (WBC *Nup*)** | **Depletion of erythrocyte cells (RBC  *Mdown*)** | **Leukocyte recovery rate (%)** | **Erythrocyte depleted rate (%)** | **Leukocyte-to-erythrocyte ratio** |
| --- | --- | --- | --- | --- | --- | --- | --- | --- |
| 1 | Female | 7.77×10^6^ | 6.61×10^9^ | 7.14×10^6^ | 5.77×10^9^ | 92% | 87% | 1:117 |
| 2 | Female | 8.28×10^6^ | 6.37×10^9^ | 7.43×10^6^ | 5.77×10^9^ | 90% | 91% | 1:75 |
| 3 | Female | 9.60×10^6^ | 7.18×10^9^ | 8.54×10^6^ | 6.34×10^9^ | 89% | 89% | 1:100 |
| 4 | Male | 9.01×10^6^ | 8.07×10^9^ | 8.45×10^6^ | 6.23×10^9^ | 93% | 90% | 1:100 |
| 5 | Female | 10.32×10^6^ | 7.62×10^9^ | 9.72×10^6^ | 7.00×10^9^ | 94% | 92% | 1:65 |
| 6 | Female | 11.30×10^6^ | 5.66×10^9^ | 10.87×10^6^ | 5.18×10^9^ | 96% | 92% | 1:48 |
| 7 | Female | 9.42×10^6^ | 6.41×10^9^ | 8.31×10^6^ | 5.61×10^9^ | 88% | 88% | 1:95 |
| 8 | Female | 7.47×10^6^ | 5.24×10^9^ | 6.62×10^6^ | 4.89×10^9^ | 87% | 93% | 1:53 |
| 9 | Female | 14.90×10^6^ | 5.01×10^9^ | 14.10×10^6^ | 4.25×10^9^ | 95% | 85% | 1:58 |
| 10 | Male | 12.47×10^6^ | 9.60×10^9^ | 11.55×10^6^ | 8.86×10^9^ | 93% | 92% | 1:62 |
| 11 | Male | 15.36×10^6^ | 8.05×10^9^ | 14.80×10^6^ | 7.25×10^9^ | 96% | 91% | 1:52 |
| 12 | Female | 9.60×10^6^ | 8.52×10^9^ | 9.10×10^6^ | 8.01×10^9^ | 95% | 94% | 1:53 |
| 13 | Male | 12.00×10^6^ | 9.71×10^9^ | 11.50×10^6^ | 8.99×10^9^ | 96% | 93% | 1:42 |
| 14 | Female | 8.26×10^6^ | 8.27×10^9^ | 7.94×10^6^ | 7.75×10^9^ | 96% | 94% | 1:42 |
| 15 | Female | 15.12×10^6^ | 9.64×10^9^ | 14.20×10^6^ | 8.92×10^9^ | 94% | 93% | 1:50 |
| 16 | Female | 11.03×10^6^ | 9.41×10^9^ | 10.02×10^6^ | 8.53×10^9^ | 91% | 91% | 1:81 |
| 17 | Female | 16.01×10^6^ | 8.91×10^9^ | 14.64×10^6^ | 8.50×10^9^ | 92% | 93% | 1:28 |
| 18 | Female | 12.69×10^6^ | 10.29×10^9^ | 11.58×10^6^ | 9.47×10^9^ | 92% | 92% | 1:68 |
| 19 | Male | 10.70×10^6^ | 9.14×10^9^ | 10.27×10^6^ | 8.23×10^9^ | 96% | 90% | 1:85 |
| 20 | Female | 9.77×10^6^ | 5.69×10^9^ | 9.62×10^6^ | 5.45×10^9^ | 98% | 96% | 1:25 |
| 21 | Female | 9.16×10^6^ | 6.65×10^9^ | 8.48×10^6^ | 6.04×10^9^ | 93% | 91% | 1:67 |
| 22 | Female | 14.70×10^6^ | 9.94×10^9^ | 13.70×10^6^ | 9.61×10^9^ | 93% | 97% | 1:23 |
| 23 | Female | 12.07×10^6^ | 9.29×10^9^ | 11.10×10^6^ | 8.72×10^9^ | 92% | 94% | 1:23 |
| 24 | Male | 19.94×10^6^ | 9.49×10^9^ | 18.92×10^6^ | 8.05×10^9^ | 95% | 85% | 1:73 |
| 25 | Male | 15.84×10^6^ | 9.68×10^9^ | 15.36×10^6^ | 8.79×10^9^ | 97% | 91% | 1:56 |
